# The Apoptosis Induction and Immunomodulating Activities of Nga-Kee-Mon (*Perilla frutescens*) Seed Extract

**DOI:** 10.3390/foods14213685

**Published:** 2025-10-29

**Authors:** Pongsathorn Dhumtanom, Anurak Wongta, Wantida Chaiyana

**Affiliations:** 1 Herbs and Functional Products Research Unit, Multidisciplinary Research Institute, Chiang Mai University, Chiang Mai 50200, Thailand; pongsathorn.d@cmu.ac.th; 2 School of Health Science Research, Research Institute for Health Sciences, Chiang Mai University, Chiang Mai 50200, Thailand; 3 Department of Pharmaceutical Sciences, Faculty of Pharmacy, Chiang Mai University, Chiang Mai 50200, Thailand; 4 Center of Excellence in Pharmaceutical Nanotechnology, Faculty of Pharmacy, Chiang Mai University, Chiang Mai 50200, Thailand; 5 Research Center of Deep Technology in Beekeeping and Bee Products for Sustainable Development Goals: SMART BEE SDGs, Chiang Mai University, Chiang Mai 50200, Thailand; 6 Multidisciplinary and Interdisciplinary School, Chiang Mai University, Chiang Mai 50200, Thailand

**Keywords:** Perilla (*Perilla frutescens*) seed extract, phytochemicals, apoptosis, DNA fragmentation, HT-29, cytokines, functional food, rosmarinic acid, luteolin

## Abstract

*Perilla frutescens*, “Nga-Kee-Mon” in Thai, is a high-nutritional-value plant. This study aims to identify the phytochemicals, apoptosis induction and immunomodulating activities of the perilla seed extract (PSE) and highlight the high pharmaceutical value of perilla. The phytochemical profile of PSE was characterized using HPLC. Antioxidant capacity was studied using DPPH assay. Apoptosis was confirmed by morphological changes and DNA fragmentation of the human colon adenocarcinoma cell line (HT-29). Immunomodulating activity was studied in an LPS-stimulated murine macrophage cell line (RAW 264.7). PSE had high levels of TPC (375.04 ± 11.45 mg GAE/g) and TFC (223.45 ± 16.02 mg QE/g) with strong radical scavenging capacity (312.87 ± 12.98 mg TE/100 g). Rosmarinic acid (0.116 g%) and luteolin (0.010 g%) were the major phytochemicals. PSE at 50 µg/mL, equivalent to 0.85 and 0.08 µg/mL of rosmarinic acid and luteolin, respectively, caused morphological alterations and DNA fragmentation within 24 h. PSE at 200 µg/mL, equivalent to 3.38 and 0.30 µg/mL of rosmarinic acid and luteolin, respectively, had significant inhibitory activity on IL-1β, IL-6, and TNF-α secretion. These results demonstrate that PSE has high antioxidant capacity, with rosmarinic acid and luteolin as the major phytochemicals. It can trigger apoptosis in HT-29 cells and has immunomodulatory effects. These findings highlight the potential of perilla seed extract as a promising natural source for therapeutic applications related to oxidative stress, cancer prevention, and immune modulation.

## 1. Introduction

*Perilla frutescens* (PF), commonly known as Nga-kee-mon in Thailand, is extensively cultivated in East and Southeast Asia [1]. The leaves are predominantly utilized in Chinese, Japanese and Korean culinary applications rather than the seeds. In Thailand, roasted seeds are traditionally ground and combined with sticky rice to create “Kao-Nug-Ngaa,” a northern Thai dish commonly found in Chiang Mai and Mae Hong Son provinces. While perilla seeds are infrequently featured in mainstream cuisine, both the oil and byproducts from seeds have attracted commercial interest for dietary supplements and animal feed components, offering a range of scientifically supported benefits that cannot be overlooked.

Numerous studies have reported on the biochemical and pharmacological properties of PF, with particular focus on its leaf extract. These include strong antioxidant, anti-inflammatory, and anticancer activities [2,3,4]. In contrast, only a few studies have investigated the seed extract [5,6]. PF leaves are rich in bioactive compounds such as rosmarinic acid, apigenin, and luteolin, which enhance their pharmacological properties [7]. Seeds possess higher lipid and essential oil content but exhibit comparable phytochemical profiles to leaves, albeit with varying quantities and proportions [8]. In addition to its antioxidant and anti-inflammatory activities, PSE exhibited multiple health-promoting effects, including restoration of gut microbial balance, enhancement of insulin function, hepatoprotective activity, protection against oxidative damage, and iron-chelating capacity [9]. Moreover, recent studies on PF seed residue demonstrated anti-diabetic and prebiotic effects, such as improved glucose metabolism, enhanced insulin secretion, and modulation of gut microbiota in diabetic rats [10]. Together, these findings highlighted the therapeutic potential of perilla seeds in supporting metabolic health and regulating inflammatory pathways [9,10].

Studies have shown that both leaf [7,8] and seed [11] extracts of PF can suppress the growth of colorectal cancer cell lines and reduce tumor progression in animal models. While the seed extract is increasingly recognized for its pharmacological value [12,13,14], investigations on its ability to induce apoptosis and modulate immune responses, particularly in the context of colorectal cancer, are still limited.

Therefore, this study investigates the apoptosis-inducing and immunomodulatory effects of *Perilla frutescens* seed extract (PSE) on HT-29 colon cancer cells and RAW 264.7 macrophages in vitro. The findings may enhance the understanding of PF seed bioactivity and support its use in daily consumption as functional foods and dietary supplements for colorectal cancer prevention.

## 2. Materials and Methods

### 2.1. Chemicals and Reagents

Ammonium chloride, 2,2-diphenyl-1-picrylhydrazyl (DPPH), dimethyl sulfoxide (DMSO), 3-(4,5-dimethylthiazol-2-yl)-2,5-diphenyl tetrazolium bromide (MTT), 2′,7′-dichlorofluorescin diacetate (DCFH-DA), 6-hydroxy-2,5,7,8-tetramethylchroman-2-carboxylic acid (Trolox), tocopherol (TC), phosphate-buffered saline (PBS), Folin–Ciocalteu’s phenol reagent, lipopolysaccharide (LPS), doxorubicin, and thiobarbituric acid were purchased from Sigma-Aldrich (St. Louis, MO, USA).

Standard phenolic compounds, including gallic acid (GA) and quercetin (Q), were also obtained from Sigma-Aldrich. Dulbecco’s Modified Eagle Medium (DMEM) was obtained from Gibco (Thermo Fisher Scientific, Waltham, MA, USA). Fetal bovine serum (FBS), penicillin–streptomycin, and trypsin–EDTA were sourced from HyClone (Logan, UT, USA). Proteinase K, K-buffer, and RNase A were purchased from Vivantis (Subang Jaya, Selangor, Malaysia). All other chemicals used were of analytical grade.

### 2.2. Plant Material and Extraction

PF seeds used in this study were purchased from a local market in Chiang Mai, Thailand.

The dried seeds were ground into fine powder and subjected to maceration in 70% ethanol at a ratio of 1:10 (*w*/*v*) and left at room temperature overnight (approximately 18–24 h). The resulting extract was filtered, concentrated under reduced pressure, and subsequently lyophilized to obtain powdered PSE. The PSE powder was preserved at –20 °C for subsequent applications. The extraction yield (%) was calculated using the following equation:

%yield = [Dry extract weight (g)/Dry starting material weight (g)] × 100(1)

### 2.3. Cell Lines and Culturing

Human colon adenocarcinoma cells (HT-29) and murine macrophage cells (RAW 264.7) were obtained from the American Type Culture Collection (ATCC, Manassas, VA, USA). Both cell lines were maintained in DMEM supplemented with 10% FBS, 100 units/mL penicillin, and 100 units/mL streptomycin. Cells were cultured at 37 °C in a humidified atmosphere incubator with 5% CO_2_.

### 2.4. High-Performance Liquid Chromatography (HPLC) Analysis

HPLC was performed to characterize the total phenolic compounds in the PSE, based on the method described by Pintha et al. [15], with slight modifications.

The extract was filtered prior to injection and analyzed using a LiChroCART RP-18e reversed-phase column (4.6 × 150 mm, 5 µm; Purospher STAR, Merck, Rahway, NJ, USA). The mobile phases consisted of solvent A (acetonitrile) and solvent B (10 mM ammonium formate buffer at pH 4.0 containing formic acid).

Gradient elution was performed under the following conditions:

- 0–5 min: 100% B (isocratic);
- 5–10 min: linear gradient from 0 to 20% A;
- 10–20 min: 20% A (isocratic);
- 20–60 min: linear gradient from 20% to 40% A.

Phenolic compounds were detected using a diode array detector (DAD) at 270, 330, 350, and 370 nm. Quantification was based on calibration curves constructed from phenolic acid standards.

### 2.5. Determination of Phenolic Compound

The total phenolic content (TPC) of the extract was determined using the Folin–Ciocalteu colorimetric method, as originally described by Singleton and Rossi [16], with slight modifications.

Briefly, 100 µL of the extract (1 mg/mL) or standard gallic acid solution at various concentrations was mixed with 7.9 mL of distilled water, 500 µL of Folin–Ciocalteu reagent, and 1.5 mL of 20% sodium carbonate (Na_2_CO_3_). The mixture was incubated at room temperature for 30 min. The absorbance was measured at 765 nm using a UV–visible spectrophotometer (Agilent 85453, Santa Clara, CA, USA).

TPC values were expressed as milligrams of gallic acid equivalents per gram of extract (mg GAE/g).

### 2.6. Determination of Flavonoid Compounds

The total flavonoid content (TFC) was determined using the aluminum chloride colorimetric assay, adapted from the method of Sembiring et al. [17], with slight modifications.

A standard calibration curve was constructed using various concentrations of quercetin standard diluted in 96% ethanol. For analysis, 50 µL of PSE (1 mg/mL) or quercetin standard was mixed with 10 µL of 10% aluminum chloride and 150 µL of 96% ethanol in a microplate. Then, 10 µL of 1 M sodium acetate was added to each well. The microplate was protected from light exposure and incubated at room temperature for 40 min.

The absorbance was measured at 415 nm using a UV–visible spectrophotometer (Agilent 85453, USA). TFC was expressed as milligrams of quercetin equivalents per gram of extract (mg QE/g). Ethanol (96%) was served as the reagent blank.

### 2.7. Determination of Antioxidant Activity Using the DPPH Radical Scavenging Assay

The antioxidant activity of PSE was evaluated based on its DPPH radical scavenging capacity, using a modified method of Brand-Williams et al. [18]. Briefly, 0.2 mL of PSE solution (1 mg/mL in ethanol) was mixed with 2.8 mL of 60 µM DPPH solution in methanol and incubated in the dark at room temperature for 30 min. The absorbance of the reaction mixture was measured at 515 nm using a UV–visible spectrophotometer.

Trolox (6-hydroxy-2,5,7,8-tetramethylchroman-2-carboxylic acid) was used as a standard antioxidant for calibration. A standard curve was constructed using Trolox solutions in the range of 0–8 µg/mL. The antioxidant activity of the extract was expressed as milligrams of Trolox equivalents (mg TE) per 100 g of dry extract.

### 2.8. Anti-Proliferation Assay

The antiproliferative activity of PSE was evaluated using the MTT assay, following the method of Skehan et al. [19], with modifications.

HT-29 cells were cultured at a density of 1 × 10^4^ cells/well in 96-well plates and incubated with PSE at concentrations ranging from 50 to 300 µg/mL for 24 h. Subsequently, 20 µL of MTT solution (5 mg/mL) was added to each well and incubated at 37 °C for 4 h. After incubation, the medium was replaced with 200 µL of acidic isopropanol to dissolve the formazan crystals. The absorbance was measured at 570 nm using a microplate reader. Doxorubicin at concentrations ranging from 20 to 200 µg/mL served as the positive control.

Untreated cells served as the control, and culture medium served as the blank. Each treatment was performed in triplicate. Cell viability (%) was calculated relative to the untreated control. The percentage of growth inhibition was calculated using the following equation [20]:

Growth inhibition (%) = 100 × [1 − (ODtest/ODcontrol)](2)

### 2.9. Morphological Changes, Acridine Orange/Ethidium Bromide Staining (AO/EB)

HT-29 cells were treated with PSE at concentrations of 50 µg/mL for 24, 48, and 72 h. Morphological changes in both treated and untreated cells were observed and documented via photomicrography.

Apoptotic features were further assessed by dual fluorescent staining using acridine orange (AO) and ethidium bromide (EB), based on the modified method of Liu et al. [21]. A staining solution (10 µL), containing 10 µg/mL each of AO and EB, was added directly to the cell cultures and incubated in the dark for 15 min. The stained cells were then visualized and photographed using a fluorescence microscope (Olympus CKX-41, Olympus Thailand, Bangkok, Thailand) at 24, 48, and 72 h post-treatment.

### 2.10. DNA Fragmentation Assay

DNA fragmentation analysis was performed according to the modified method of Ha et al. [22]. HT-29 cells were cultured at a density of 1 × 10^6^ cells/mL in 6-well plates and incubated at 37 °C in a 5% CO_2_ atmosphere until reaching approximately 80% confluency. The cells were then treated with PSE at concentrations of 50 µg/mL for 24 h. After treatment, cells were harvested and washed with ice-cold PBS (137 mM NaCl, 2.7 mM KCl, 10 mM Na_2_HPO_4_, and 1.8 mM KH_2_PO_4_).

The pelleted cells were lysed in cold lysis buffer (20 mM Tris-HCl, pH 7.5; 150 mM NaCl; 1 mM Na_2_EDTA; 1% Triton X-100) at 4 °C for 10 min, followed by centrifugation at 14,000 rpm for 20 min at 4 °C. The supernatant was sequentially incubated with 2 µL of RNase A (10 mg/mL) and 10 µL of proteinase K (5 mg/mL) at 37 °C for 1 h.

DNA was precipitated by adding ice-cold isopropanol at twice the volume of the sample, followed by overnight incubation at 4 °C. The DNA was then collected by centrifugation (14,000 rpm, 20 min, 4 °C), air-dried, and dissolved in 0.5× TE buffer.

DNA fragmentation was analyzed by electrophoresis on a 1.6% agarose gel containing ethidium bromide and visualized under UV illumination.

### 2.11. Inflammatory Cytokine Production

RAW 264.7 murine macrophage cells were cultured at 5 × 10^5^ cells/well in 6-well plates and cultured in DMEM supplemented with LPS (1 µg/mL) for 24 h to induce an inflammatory response. After incubation, cells were washed twice with PBS and then treated with various concentrations of PSE (50–200 µg/mL) for an additional 24 h.

Following treatment, the culture media were collected, and the concentrations of interleukin-6 (IL-6), interleukin-1β (IL-1β), and tumor necrosis factor-alpha (TNF-α) were measured using enzyme-linked immunosorbent assay (ELISA) kits (Abcam, Cambridge, UK) according to the manufacturer’s instructions.

### 2.12. Statistical Analysis

All experimental data were expressed as mean ± standard deviation (SD). Statistical analyses were performed using GraphPad Prism software (GraphPad Software, Version 4.01, San Diego, CA, USA). One-way analysis of variance (ANOVA) was conducted to determine significant differences among experimental groups. A *p*-value of less than 0.05 was considered statistically significant, and *p* < 0.01 was considered highly significant.

## 3. Results

### 3.1. Extraction Yield, Total Phenolic and Flavonoid Contents, Antioxidant Capacity, and Phytochemical Profile

Extraction using 70% ethanol from *P. frutescens* seeds yielded 68.54 ± 7.13 g of lyophilized extract per kilogram of dried seeds, corresponding to an extraction efficiency of 6.85 ± 0.71%. The antioxidant capacity of the extract, as assessed using the DPPH assay, was 312.87 ± 12.98 mg Trolox equivalents per 100 g of dry extract.

The TPC of the extract was 375.04 ± 11.45 mg GAE/g, while the TFC was 223.45 ± 16.02 mg QE/g.

HPLC analysis revealed several phenolic and flavonoid constituents. Rosmarinic acid was the predominant compound, with a concentration of 1157.36 ± 55.66 μg/g dry weight. Among the flavonoids, luteolin (103.28 ± 2.71 μg/g) and apigenin (27.45 ± 1.12 μg/g) were the most abundant. Minor constituents included gallic acid, quercetin, caffeic acid, tannic acid, isoquercetin, apigenin, and catechin, each present at concentrations below 30 μg/g. A detailed summary of phytochemical composition is provided in Table 1.

### 3.2. HPLC Analysis of Phenolic Compounds

**Figure 1:**
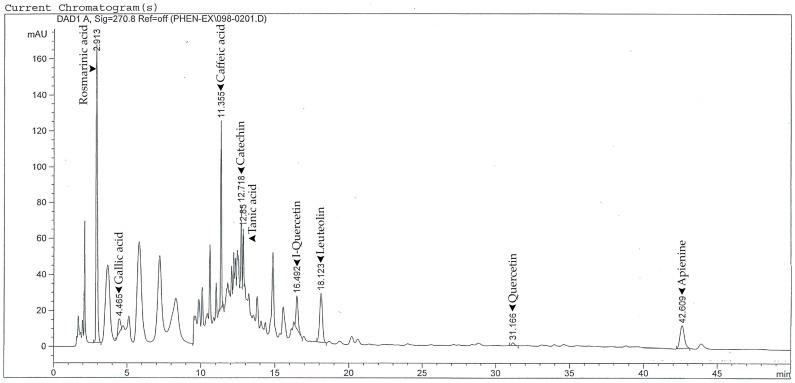
HPLC chromatogram of PSE, showing major phenolic compounds. Rosmarinic acid was the predominant compound, followed by tannic acid, quercetin, and I-quercetin. Peaks were detected at 270–370 nm.

The chromatographic profile of PSE obtained by HPLC is shown in Figure 1. The dominant peak corresponded to rosmarinic acid, representing 0.116 g% of the extract. Minor peaks were also detected for luteolin (0.010 g%), apigenin (0.003 g%), and gallic acid (0.002 g%).

These findings confirm that rosmarinic acid is the predominant phenolic compound in PSE, which is consistent with previous reports on Perilla species. Despite their lower concentrations, apigenin and luteolin may still contribute to the extract’s biological effects observed in later assays.

### 3.3. Antiproliferative Effect of PSE on HT-29 Cells

**Figure 2:**
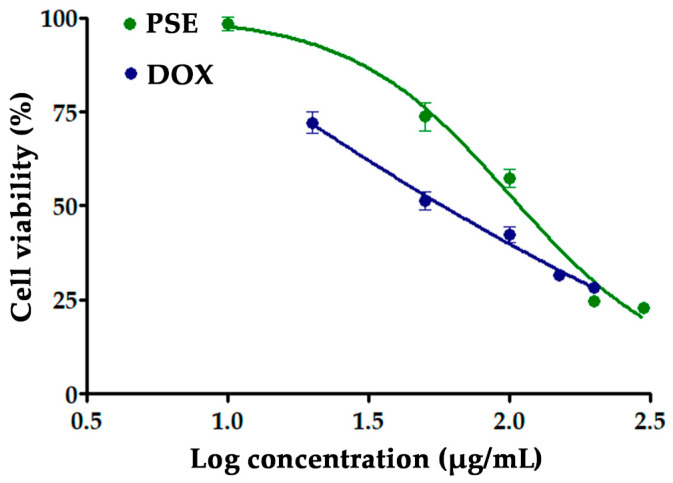
Dose–response curve of doxorubicin (DOX) and PSE on HT-29 cell viability.

The cytotoxic effect of PSE on HT-29 human colon cancer cells was assessed using the MTT assay. After 24 h of exposure, PSE induced a concentration-dependent reduction in cell viability (Figure 2). The half-maximal inhibitory concentration (IC_50_) was calculated to be 103.91 ± 17.20 µg/mL, compared to 0.86 ± 0.42 µg/mL for doxorubicin. While PSE was less potent than the standard chemotherapeutic agent, it still exerted significant cytotoxic effects at higher concentrations.

Treatment with PSE at concentrations ranging from 50 to 300 µg/mL significantly reduced cell viability compared with the untreated control (*p* < 0.0001), as determined by one-way ANOVA with Tukey’s post hoc test. No significant difference was observed between the 200 and 300 µg/mL groups, suggesting a cytotoxicity plateau at higher concentrations.

### 3.4. Morphological Changes and Fluorescence Staining Cell

Morphological changes and AO/EB staining of HT-29 cells were observed under light transmission and fluorescence microscopy (Olympus CKX-41), respectively. Staining with the fluorescent dyes AO/EB was used to differentiate between live, apoptotic, and necrotic cells based on their membrane integrity and nuclear morphology. Live cells stained only with AO appeared green; in contrast, apoptotic cells, stained with both AO and EB (which penetrates cells with compromised membranes), appeared bright green or a mix of green and orange due to nuclear condensation and fragmentation. Necrotic cells appeared orange or red due to EB staining following dye uptake. Upon treatment with 50 μg/mL of PSE, HT-29 cells exhibited morphological features typical of apoptosis within 72 h, including cell shrinkage, membrane blebbing, chromatin condensation, nuclear fragmentation, and formation of apoptotic bodies.

**Figure 3:**
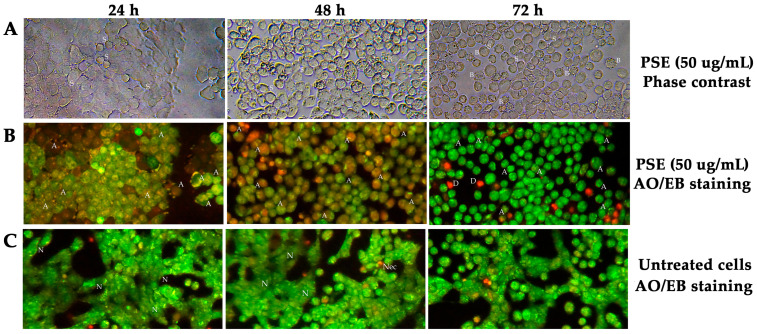
Morphological changes (**A**) and AO/EB staining of HT-29 (**B**,**C**) treated with 50 µg/mL PSE were demonstrated. Morphological changes and AO/EB staining of HT-29 cells (**A**,**B**) and untreated HT-29 (**C**) were observed under light transmission and a fluorescent microscope (Olympus CKX-41), respectively. N, normal; A, apoptotic bodies; Nec, necrotic cell; S, shrinkage; B, blebbing cell; and D, death cell. AO/EB staining indicates cell viability and death, with viable cells fluorescing green, late apoptotic cells showing orange to red fluorescence, and necrotic cells displaying red staining.

Cellular morphological changes were observed using light microscopy. An increasing incubation period (Figure 3A) resulted in cell shrinkage, cell size reduction, sloughing and increased cell death. Chromatin condensation and blebbing were observed within 24 h following AO-EB staining of PSE-treated HT-29 cells (Figure 3B). AO/EB-stained untreated HT-29 cells (negative control) are shown in Figure 3C.

### 3.5. DNA Fragmentation in HT-29 Cells

**Figure 4:**
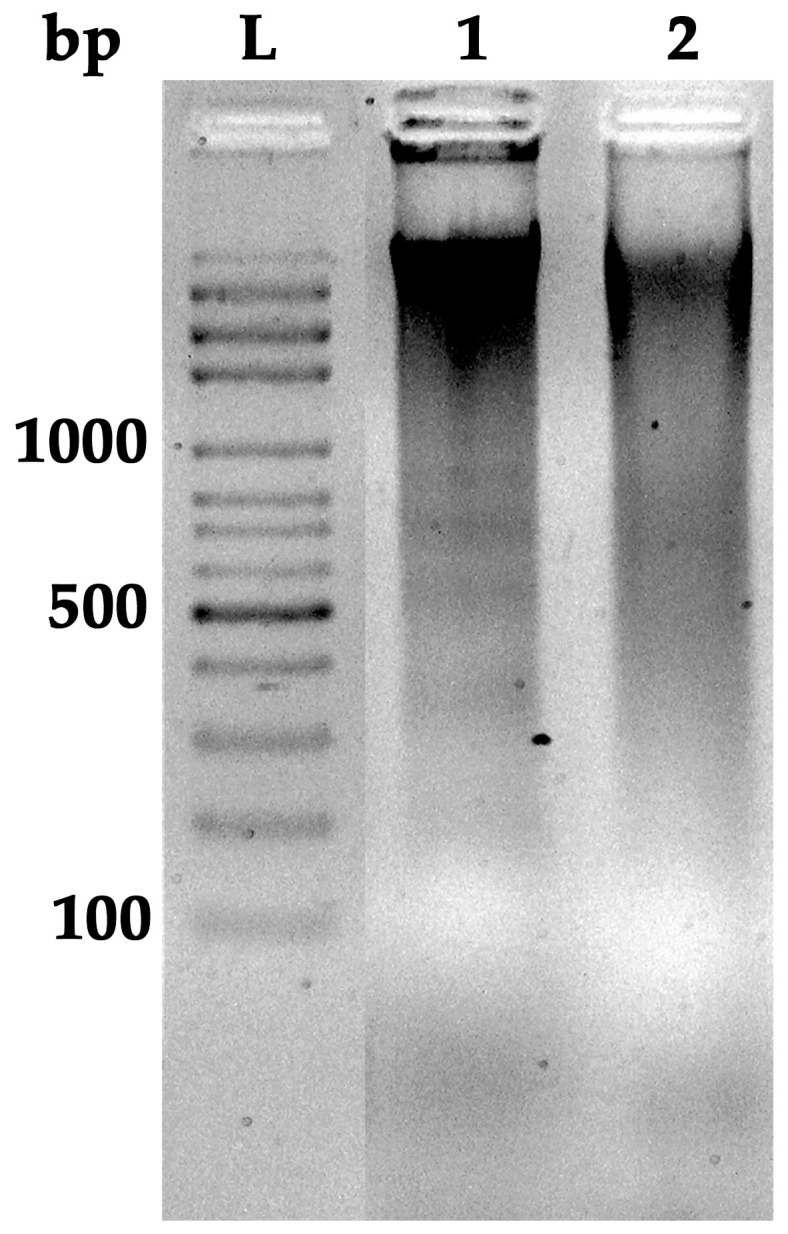
DNA fragmentation detected by agarose gel electrophoresis in HT-29 cells following treatment with 50 μg/mL PSE for 24 h (lane 1). Lane 2: untreated control; Lane L: 100 bp DNA ladder. Image contrast and brightness were adjusted using Adobe Photoshop CS6.

To investigate whether the reduction in HT-29 cell viability was linked to apoptosis, DNA fragmentation was assessed by agarose gel electrophoresis. As shown in Figure 4, lane L represents a 100 bp DNA ladder used as a molecular size marker for reference. The untreated control (lane 2) displayed a single, high-molecular-weight DNA band near the top of the gel, indicating intact genomic DNA and the absence of fragmentation, which is characteristic of viable, non-apoptotic cells. In contrast, treatment with 50 µg/mL PSE for 24 h (lane 1) induced characteristic DNA laddering pattern, represented by a series of regularly spaced DNA fragments of approximately 180–200 bp and their multiples. This oligonucleosomal fragmentation pattern is indicative of apoptosis, reflecting internucleosomal cleavage of chromatin by activated endonucleases during the late stages of programmed cell death.

These observed fragmentations in PSE-treated cells confirmed that PSE induced apoptosis in HT-29 cells through DNA fragmentation, even at moderate concentrations and short exposure times. This is consistent with the morphological changes observed in AO/EB staining (Section 3.4), such as chromatin condensation and nuclear fragmentation, further confirming the pro-apoptotic effect of the extract. Therefore, these findings suggest that PSE-mediated cytotoxicity in HT-29 cells is primarily attributable to apoptosis.

### 3.6. Effect on Pro-Inflammatory Cytokine Secretion

Stimulation of RAW 264.7 macrophages with LPS (1 µg/mL) markedly increased the secretion of pro-inflammatory cytokines. Compared with the untreated control, IL-6, IL-1β, and TNF-α levels rose to 237.05 ± 2.88, 271.08 ± 2.57, and 713.90 ± 2.81 pg/mL, respectively (*p* < 0.0001).

**Figure 5:**
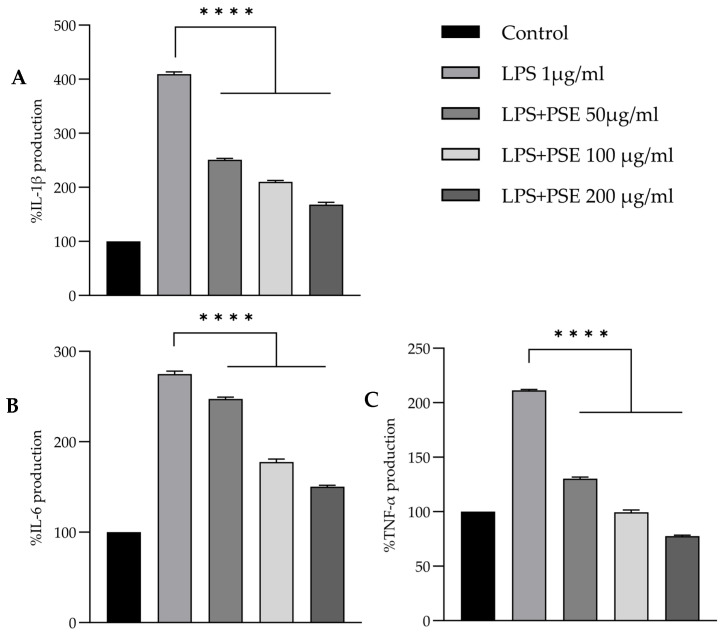
Dose-dependent inhibition of IL-6 (**A**), IL-1β (**B**), and TNF-α (**C**) secretion by PSE in LPS-stimulated RAW 264.7 macrophages. Cytokine levels were quantified by ELISA after 24 h incubation. Values represent mean ± SD (*n* = 3). Statistical significance was determined by one-way ANOVA followed by Tukey’s post hoc test. **** *p* < 0.0001 vs. LPS-only group.

Co-treatment with PSE (50, 100, and 200 µg/mL) for 24 h significantly and dose-dependently reduced the secretion of all three cytokines (Figure 5A–C). At the highest concentration (200 µg/mL), IL-6, IL-1β, and TNF-α levels decreased to 129.59 ± 1.30, 111.32 ± 2.75, and 262.24 ± 2.58 pg/mL, representing reductions of approximately 45%, 59%, and 63%, respectively, compared with the LPS-only group.

These results demonstrate that PSE suppresses LPS-induced pro-inflammatory cytokine production in macrophages, supporting its potential immunomodulatory activity.

## 4. Discussion

Unlike the leaves, which are not commonly used in Thai cuisine, *P. frutescens* seeds have traditionally been consumed in northern Thailand as snacks, often mixed with sticky rice. This contrasts with culinary practices in Korea and China, where the leaves are widely incorporated into soups and grilled dishes. In Thailand, interest in both the seeds and leaves has increased in recent years, partly driven by the influence of Korean popular culture. Although current consumption remains limited, the medicinal potential of *P. frutescens* and its status as a low-contamination crop in northern Thailand highlight its promise as a functional food ingredient and as a candidate for geographical indication (GI) promotion.

Phytochemical analyses and previous studies have reported diverse pharmacological activities of *P. frutescens*, including antioxidant, anti-inflammatory, anti-allergic, antibacterial, antifungal, and anticancer properties [22,23,24,25,26,27,28,29,30,31,32,33,34,35,36]. Our HPLC analysis showed that the seed extract contains rosmarinic acid, luteolin, apigenin, and quercetin, a profile comparable to that of other medicinal herbs such as *Ocimum* species. These findings provide a rationale for further investigation into its biochemical effects, particularly in immune modulation and apoptosis induction.

*P. frutescens* leaves have been found to be rich in phenolic and flavonoid components [37], which are important secondary metabolites of many plants, and exhibit a variety of biochemical and biological activities [38]. The 70% ethanol extract of PSE yielded 6.85% (*w*/*w*). Quantitative analysis showed a high concentration of rosmarinic acid (0.116 g%) and a lower amount of luteolin (0.010 g%), along with minor constituents such as apigenin and quercetin. These levels were higher than those reported from seed residue by Chantana et al. [11] and comparable to the concentrations found in fresh leaves as described by Tantipaiboonwong et al. [39]. Leaves and seeds are important contributors of phenolic compounds; yet, making direct comparisons is challenging because of the variability found in plant parts, farming practices, and extraction methods.

Phenolic compounds exert antioxidant activity primarily through metal ion chelation and inhibition of radical-producing enzymes such as cytochrome P450s, lipoxygenases, and xanthine oxidase [40,41]. Flavonoids, including luteolin, have also been reported to display anti-inflammatory, antimicrobial, cytotoxic, and antitumor effects. These biological activities are often mediated by modulation of signal transduction pathways, regulation of apoptosis-related genes, and suppression of pro-inflammatory cytokines [2,42].

In the current study, the antioxidant activity of PSE was evaluated using the DPPH radical scavenging assay. DPPH is a stable free radical, and its scavenging by antioxidants reflects the ability of the extract to donate electrons or hydrogen atoms, thereby neutralizing reactive species [43]. Mechanistically, such antioxidant activity can mitigate oxidative stress, which is closely linked to the regulation of apoptosis: excessive reactive oxygen species can trigger mitochondrial dysfunction, upregulate pro-apoptotic factors (e.g., Bax, caspase-3), and downregulate anti-apoptotic proteins (e.g., Bcl-2) [44]. By reducing ROS levels, PSE may help modulate these apoptotic pathways, contributing to its potential cytoprotective or anticancer effects. While DPPH provided valuable insight into free radical scavenging, it is only one aspect of antioxidant activity. Incorporating additional assays that evaluate different antioxidant mechanisms would provide a more comprehensive assessment of the antioxidant potential of PSE. For example, the 2,2′-azino-bis(3-ethylbenzothiazoline-6-sulfonic acid) (ABTS^•+^) radical cation scavenging assay evaluates radical scavenging in both aqueous and lipid environments, providing a broader assessment of antioxidant capacity [45]. The ferric reducing antioxidant power (FRAP) assay measures electron-donating (ferric-reducing) ability [46], while the oxygen radical absorbance capacity (ORAC) assay determines the capacity to neutralize peroxyl radicals, reflecting chain-breaking antioxidant activity [47]. Incorporating these complementary assays is suggested to offer a more comprehensive understanding of the antioxidant potential of PSE and its possible link to apoptotic and immunomodulatory effects. However, the DPPH inhibition observed in the current study suggests that *P. frutescens* seed would be a valuable source of bioactive compounds with the potential to induce biological responses such as apoptosis and immune modulation.

In our study, PSE at 50 µg/mL, equivalent to approximately 0.85 µg/mL rosmarinic acid and 0.08 µg/mL luteolin, induced chromatin condensation and DNA fragmentation in HT-29 human colon cancer cells within 24 h, demonstrating its antiproliferative potential and suggesting apoptosis induction. The present findings demonstrate that PSE exerts both antitumor and anti-inflammatory effects through its bioactive constituents. The induction of chromatin condensation and DNA fragmentation in HT-29 colorectal cancer cells following treatment with 50 µg/mL PSE indicates the activation of apoptosis pathways, consistent with antiproliferative mechanisms that suppress cancer cell survival [48,49]. This observation suggests that PSE may inhibit tumor progression by promoting programmed cell death rather than necrosis, thereby minimizing inflammatory responses often associated with cancer cell lysis [50]. The observed concentrations of rosmarinic acid and luteolin, although relatively low, were sufficient to elicit a marked cellular response, highlighting the potency and synergistic interactions of these phytochemicals within the extract matrix. Luteolin has been reported to induce G1 phase cell cycle arrest and apoptosis in HT-29 cells by reducing DNA synthesis and modulating pro-apoptotic proteins [51]. Rosmarinic acid derived from *Rubus chingii* induces apoptosis in gastric and liver cancer cells via mitochondrial signaling pathways, influencing Bcl-2, Bax, cytochrome C, and caspase-3 [52]. However, studies also indicate that crude extracts may outperform pure compounds in biological activity, possibly due to synergistic interactions among multiple phytochemicals [4,22].

In addition to apoptosis induction, PSE also significantly attenuated the secretion of pro-inflammatory cytokines IL-1β, IL-6, and TNF-α in LPS-stimulated macrophages, supporting its strong anti-inflammatory activity. This dual action, both apoptosis induction in cancer cells and suppression of inflammatory mediators in macrophages, suggests that PSE may act through a coordinated mechanism that both limits tumor cell proliferation and modulates the tumor microenvironment to reduce chronic inflammation, a known driver of colorectal carcinogenesis [53,54]. These results align with previous in vivo findings by Chantana et al. [11], where PF seed residue extract suppressed aberrant crypt foci formation, but the present study extended the understanding by providing cellular-level evidence of apoptosis and cytokine modulation. Mechanistically, the suppression of pro-inflammatory cytokines (IL-1β, IL-6, and TNF-α) by PSE in LPS-stimulated macrophages may involve the inhibition of the nuclear factor kappa-B cells (NF-κB) and mitogen-activated protein kinase (MAPK) signaling pathways, which are key regulators of inflammatory responses [55,56,57]. Luteolin and rosmarinic acid, the major phenolic constituents of PSE, have been reported to inhibit inhibitor of nuclear factor kappa B alpha (IκBα) phosphorylation and p65 nuclear translocation, thereby reducing NF-κB-mediated transcription of pro-inflammatory genes [58,59]. Moreover, activation of the nuclear factor erythroid 2-related factor 2 (Nrf2)/heme oxygenase-1 (HO-1) antioxidant pathway by phenolic compounds could contribute to the observed immunomodulatory effect by reducing oxidative stress-induced inflammation [60]. Therefore, these findings emphasize the novel potential of PSE as a multifunctional natural agent with both antiproliferative and anti-inflammatory properties relevant to colorectal cancer prevention and therapy, along with the potential to maintain immune homeostasis.

Additionally, these findings indicated that the effects of PSE resulted from a synergistic interaction of multiple constituents rather than from rosmarinic acid or luteolin alone. The observed antiproliferative and pro-apoptotic effects of PSE may be partly attributed to the synergistic interactions between rosmarinic acid and luteolin, two of its major bioactive constituents. Previous studies have reported that co-treatment with rosmarinic acid and luteolin exerted a pronounced synergistic effect in cancer cells, including HT-29 colorectal cancer cells [61]. Specifically, the combination enhanced intracellular reactive oxygen species scavenging and upregulated antioxidant enzymes such as superoxide dismutase and catalase, compared with either compound alone [61]. At the same time, rosmarinic acid and luteolin significantly increased the Bax/Bcl-2 ratio and activated caspase-3 and caspase-9, leading to apoptotic cell death [23,62]. These results suggest that rosmarinic acid and luteolin act synergistically to enhance cytoprotective antioxidant responses and pro-apoptotic mechanisms, likely through coordinated modulation of oxidative stress and inflammatory signaling pathways [23,62,63,64]. The combined polyphenolic treatment not only suppresses NF-κB activation and pro-inflammatory cytokine release in LPS-stimulated macrophages [63] but also promotes Nrf2/HO-1 pathway activation, as demonstrated in neuroprotective studies using the human neuroblastoma cell line (SH-SY5Y) [64], highlighting the multifaceted synergistic potential of these compounds. In the context of PSE, the coexistence of these compounds may therefore amplify its antitumor and anti-inflammatory potential, supporting the idea that crude extracts can outperform individual phytochemicals due to such synergistic interactions. The evidence suggests that whole-seed extracts may offer enhanced therapeutic effects compared to isolated phytochemicals. In recent years, several studies have examined *P. frutescens* leaf extracts [23,29,36,65,66,67,68,69], but few have focused on the seed. Our study contributes to this less explored area by providing evidence that PSE induces apoptosis in cancer cells and suppresses pro-inflammatory cytokines in macrophages, likely due to its high phenolic and flavonoid content. Further evaluation of apoptosis-related genes (Bcl-2, Bax, caspase-3) by polymerase chain reaction (PCR) or their protein levels by ELISA could provide valuable mechanistic insight into PSE-induced apoptosis and is therefore recommended for future studies to further elucidate its molecular pathways.

These findings indicate that *P. frutescens* seed extract possesses anticancer and anti-inflammatory attributes. Subsequent research should examine its use in dietary supplements or functional foods, along with in vivo efficacy and safety assessments. Further investigation is required to analyze the bioactivity of specific phytochemicals relative to the crude extract to elucidate synergistic mechanisms and inform product development.

## 5. Conclusions

This study demonstrated that PSE exerted significant antitumor effects through its antiproliferative and pro-apoptotic activity in HT-29 human colon cancer cells while also displaying anti-inflammatory and immunomodulatory effects in LPS-stimulated macrophages. Notably, PSE treatment led to chromatin condensation and DNA fragmentation, which strongly supported its ability to suppress cancer cell growth via apoptosis, highlighting its antiproliferative mechanism. In addition, the antioxidant activity observed in the DPPH assay may further contribute to its apoptosis-inducing and immunomodulatory properties, suggesting a coordinated mechanism by which PSE modulates both tumor cells and the inflammatory microenvironment. The present findings are both significant and novel, providing cellular-level evidence of dual anticancer and anti-inflammatory activities of PSE. Notably, the observed antiproliferative effects further highlight the therapeutic potential of PSE, as it directly inhibited cancer cell growth while also influencing inflammatory processes. To further elucidate the molecular mechanisms underlying these effects and support potential therapeutic or dietary applications, future studies should examine apoptosis- and proliferation-related genes and proteins (e.g., Bcl-2, Bax, caspase-3, Ki-67), compare the bioactivity of individual phytochemicals versus the crude extract to clarify synergistic interactions, and conduct in vivo efficacy and safety assessments. Beyond preclinical studies, the results highlighted the potential of PSE as a functional food ingredient, suggesting its incorporation into dietary strategies for colorectal cancer prevention or as a natural adjunct to conventional therapies.

## Figures and Tables

**Table 1:** Bioactive compounds and antioxidant-related properties of *P. frutescens* seed extract.

Parameter	Value (Mean ± SD)	Unit	% *w*/*w*
Extraction of PF seed by 70% ethanol	6.85 ± 0.71	g from 100 g of dried weight	6.85 g%
Antioxidant capacity of PSE using DPPH assay	312.87 ± 12.98	mg eq.Trolox/100 g of dry extract	-
Total phenolic contents	375.04 ± 11.45	mg GAE/g extract	37.5 g%
Total flavonoid contents	223.45 ± 16.02	mg QE/g extract.	22.4 g%
Rosmarinic acid	1157.36 ± 55.66	µg/g dry weight	0.116 g%
Luteolin	103.28 ± 2.71	µg/g dry weight	0.010 g%
Apigenin	27.45 ± 1.12	µg/g dry weight	0.003 g%
Gallic acid	24.89 ± 1.93	µg/g dry weight	0.002 g%
Quercetin	22.22 ± 1.18	µg/g dry weight	0.002 g%
Caffeic acid	21.30 ± 1.09	µg/g dry weight	0.002 g%
Tannic	16.69 ± 0.92	µg/g dry weight	0.0016 g%
I-Quecetin	14.32 ± 0.82	µg/g dry weight	0.0014 g%
Catechin	6.06 ± 0.51	µg/g dry weight	0.0006 g%

## Data Availability

The original contributions presented in the study are included in the article; further inquiries can be directed to the corresponding author.

## Abbreviations

The following abbreviations are used in this manuscript:

ABTS^•+^	2,2′-Azino-bis(3-ethylbenzothiazoline-6-sulfonic acid radical
AO	Acridine orange
AO/EB	Acridine orange/Ethidium bromide staining
ANOVA	One-way analysis of variance
DCFH-DA	2′,7-dichlorofluorescin diacetate
DAD	Diode array detector
DMSO	Dimethyl sulfoxide
DMEM	Dulbecco’s Modified Eagle Medium
DPPH	2,2-diphenyl-1-picrylhydrazyl
EB	Ethidium bromide
FBS	Fetal bovine serum
FRAP	Ferric reducing antioxidant power
GA	Gallic acid
GI	Geographical indication
HO-1	Heme oxygenase-1
HT-29	Human colon adenocarcinoma cells
IC_50_	Half-maximal inhibitory concentration
IκBα	Inhibitor of nuclear factor kappa B alpha
IL-1β	Interleukin-1β
IL-6	Interleukin-6
MAPK	Mitogen-activated protein kinase
MTT	3-(4,5-Dimethylthiazol-2-yl)-2,5-diphenyl tetrazolium bromide
NF-κB	Nuclear factor kappa-B cells
Nrf2	Nuclear factor erythroid 2-related factor 2
ORAC	Oxygen radical absorbance capacity
PBS	Phosphate-buffered saline
PCR	Polymerase chain reaction
PF	*Perilla frutescens*
PSE	*Perilla frutescens* seed extract
Q	Quercetin
RAW 264.7	Murine macrophage cells
SD	Standard deviation
SH-SY5Y	Human neuroblastoma cell line
TC	Tocopherol
TFC	Total flavonoid content
TPC	Total phenolic content
TNF-α	Tumor necrosis factor-alpha
Trolox	6-hydroxy-2,5,7,8-tetramethylchroman-2-carboxylic acid
